# In Situ Probing the Relaxation Properties of Ultrathin Polystyrene Films by Using Electric Force Microscopy

**DOI:** 10.1186/s11671-017-2019-7

**Published:** 2017-04-07

**Authors:** Xiaoqin Qian, Zihong Lin, Li Guan, Qiang Li, Yapei Wang, Meining Zhang, Mingdong Dong

**Affiliations:** 1 Department of Chemistry, Renmin University of China, 100872 Beijing, China; 2 Interdisciplinary Nanoscience Center (iNANO), University of Aarhus, DK-8000 Aarhus C, Denmark

**Keywords:** Glass transition temperature, Ultrathin films, Surface potential, Electric force microscopy

## Abstract

The rapid development of nanoscience and nanotechnology involves polymer films with thickness down to nanometer scale. However, the properties of ultrathin polymer films are extremely different from that of bulk matrix or thin films. It is challenging to distinguish the changes of physical properties in ultrathin films using conventional techniques especially when it locates near the glass transition temperature (*T*_g_). In this work, we successfully evaluated a series of physical properties of ultrathin polystyrene (PS) films by in situ characterizing the discharging behavior of the patterned charges using electric force microscopy. By monitoring the surface potential in real time, we found that the *T*_g_ of ultrathin PS films is clearly independent of film thickness, which are greatly different from that of thin PS films (film thickness larger than 10 nm).

## Background

Thin polymer films display distinct differences from their bulk matrix, including those related to crystallization [1, 2], physical cross-linking in polymers [3], thermal expansion coefficients [4–7], and physical aging [8–12]. Decreasing the thickness of thin films below 10 nm, the physical and chemical properties of ultrathin films are significantly different in comparison with thin films due to the thickness confinement effect [13–15]. To explore such a thickness-dependent effect, a great deal of effort has been devoted to investigating the molecular mobility and relaxation dynamics within ultrathin polymer films [14, 16–18]. Previous studies have revealed that the surface relaxation dynamics in ultrathin films were apparently different from that in bulk materials and normal thin films [19, 20]. The glass transition temperature (*T*_g_) of ultrathin films was far below the bulk *T*_g_ due to the enhanced mobility near the free surface [13, 21–24]. For instance, the *T*_g_ of normal thin polystyrene (PS) films deposited on silicon substrate was found to drop by 20 K from the bulk *T*_g_, while the *T*_g_ almost dropped 70 K for freely standing thin PS films [25]. A high surface-to-volume ratio, in which the ultrathin films are dominated by the surface properties, can considerably affect the relaxation dynamics near the interface [26–29].

The anomalous thermal behavior observed in thin films is closely related to the presence of surface and interfaces [10]. Two models [7, 11, 12, 22, 30–33], named as the three-layer model and the two-layer model, have been widely used to explain the decrease of *T*_g_ for nano-confined polymer films. In the three-layer model, the interfacial layer, which is also described as the dead layer, plays a great role in the relaxation behavior [12, 34], and the conformation of the polymer chain within interfacial layer is affected by the polymer-substrate interaction. Polymer chains in the middle layer are confined in the matrix, exhibiting the intrinsic characteristics of the polymer matrix assumed to be the bulk *T*_g_ [32].When the film thickness decreases to several nanometers, the interfacial layer has disappeared; the thin polymer film then could be illustrated by the two-layer model. In the two-layer model, the top layer, which is also regarded as a liquid-like layer, is supposed to enhance the mobility of polymer chains and induce the decrease of *T*_g_ [29].

Various kinds of techniques have been attempted to quantitatively measure the *T*_*g*_ of ultrathin polymer films [11, 18, 32, 34–38]. Particularly, the molecular motion and relaxation dynamics in ultrathin polymer films have been extensively investigated by many groups using some intriguing techniques [18]. By using X-ray reflectivity, Tsukasa et al. found that the *T*_g_ of ultrathin films was almost independent of film thickness when it fell below 10 nm [18]. Keddie measured the *T*_g_ of ultrathin PS films with the thickness less than 20 nm using ellipsometry and found that the *T*_g_ was depressed from the bulk values. [7] However, it is still challenging to investigate the relaxation dynamics and *T*_g_ shifts in ultrathin films, since the differences in properties near *T*_g_ become rather smaller when using the traditional techniques as stated above [39]. Increasing attention is then paid to atomic force microscopy (AFM) method, which has the advantages of systematically measuring relaxation dynamics based on the mechanical and electrical characteristics simultaneously with the observation of surface morphologies. For example, Akabori et al. demonstrated how the surface relaxation behavior depended on the thickness of PS films using lateral force microscopy (LFM) [10]. Thermal molecular motion at the outermost surface of the PS film was directly measured using scanning viscoelasticity microscopy (SVM) [35]. The *T*surf g, which is defined as the glass transition temperature at the surface, decreased compared to the bulk *T*bulk g [40].

In this work, patterned charges were introduced onto the ultrathin PS films (less than 10 nm) by selectively charging with a polydimethylsiloxane (PDMS) stamp. The surface potential decay (SPD) of the patterned charges is in situ monitored using electric force microscopy (EFM). Compared to traditional methods, the discharging behavior is more sensitive to the very small-scale motion of polymer chains, especially in the ultrathin films, and the discharging rate is closely related to the relaxation status of the polymer films. Hence, the relationship between relaxation dynamics and the behavior of the charge decay is identified in ultrathin PS films, which is also proved to be greatly different from that of normal thin PS films. The direct observation of charge decay behaviors in ultrathin polymer films provides a more sensitive approach to study the relaxation and glass transition behaviors of polymer chains, and to quantify a series of physical properties in ultrathin films.

## Methods

### Materials

All materials and chemicals are commercially available and used as received. PS (Mw = 4000) was purchased from Alfa Aesar, and chlorobenzene was purchased from Sinopharm Chemical Reagent Beijing Co. The single-side polished silicon wafer (<100>) doped with phosphorus (2 − 4 Ω cm) was purchased from Silicon Quest International. Polydimethylsiloxane (PDMS, Sylgard 184, Dow Corning Co.) was used to fabricate the PDMS stamp.

### Fabrication of Charge Patterns

Ultrathin PS films were prepared by spin-casting PS solution on silicon wafers. The thickness of the ultrathin PS films was determined by changing the concentration of PS solution in chlorobenzene and the spin-coating speed. Charge patterns were generated on the ultrathin PS films using an electric microcontact printing (e-*μ*CP) technique [37]. A micro-patterned PDMS stamp was needed to inject charges in the specific areas on ultrathin film [36].

**Fig. 1:**
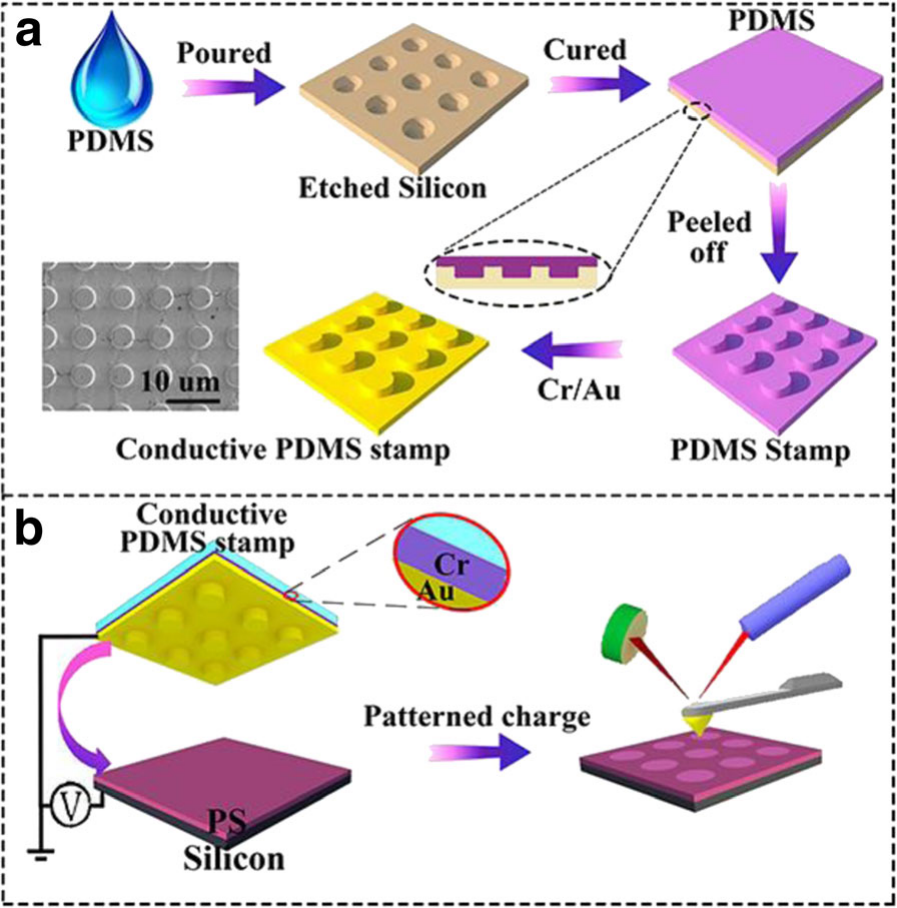
**a** Schematic illustration of the fabrication of PDMS stamps and **b** the in situ monitoring of discharging behavior

The schematic fabrication processes of PDMS stamps and patterned charges were illustrated in Fig. 1. PDMS prepolymer was poured on an optical lithographed silicon wafer with micro-structures and cured at a temperature of 348 K for 2 h. Then, the cured PDMS polymer was peeled off from the silicon substrate, and a 10-nm Cr adhesive layer and 100 nm Au conductive layer was deposited on it, forming a conductive micro-patterned PDMS stamp. The conductive PDMS stamp was then placed in close contact with the PS film supported on Si substrate to form a parallel-plate capacitor. A Keithley 2400 source meter supplied a 10 kV cm^−1^ electrical field between the PDMS stamp and the silicon substrate. The charge patterns were successfully fabricated and characterized using EFM.

### SPD Measurement

All topographies and surface potential images in this study were recorded using a Dimension Icon system (Bruker, USA) with an in situ heater/cooler accessory. A low relative humidity between 20% and 30% was controlled by trial and error to ensure that the patterned charges were stored in a stable status. The charges decay properties were characterized in situ using EFM. A 30 μm × 30 μm area was scanned to obtain sufficient patterns for reliable statistics. The measurements were conducted at a constant heating rate of 2 K/min starting from the room temperature (298 K) to the desired temperature. An adequate waiting time of 3 min and a temperature interval of 10 K are adopted to avoid possible measuring errors by trial and error. The surface potential was measured, and the histograms of the captured data were counted to get accurate and reliable results.

## Results and Discussion

**Fig. 2:**
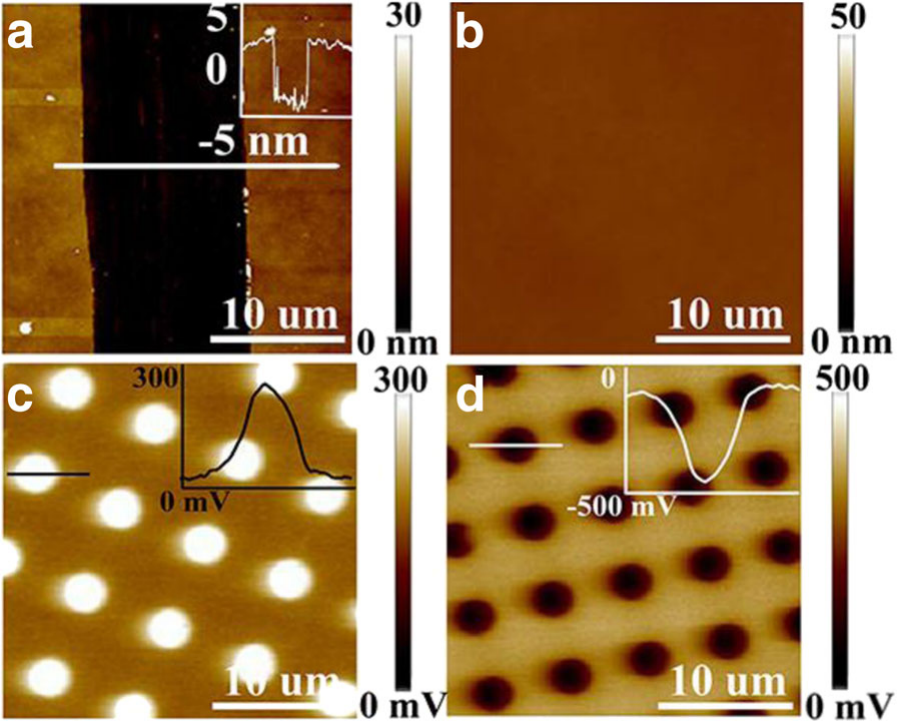
AFM topography images and surface potential of ultrathin PS films obtained in a single measurement. **a** The scratched film for thickness measurement. **b** AFM morphology of charged ultrathin PS film. **c** Surface potential images of charged ultrathin PS film with positive charge and **d** negative charge

Charge patterns in accord with the electrode template are successfully fabricated as illustrated in Fig. 1b. The ultrathin PS film is scratched to create some grooves for the measurement of film thicknesses (Fig. 2a). As an excellent polymer electret, PS film is able to store electric charges for a long time. It should be noted that only the areas contacted by the stamp can be electrically injected. Charge patterns are successfully fabricated without any morphological deformation, as shown in Fig. 2b. Both positive and negative charges with uniform patterns could be successfully injected onto the ultrathin PS films, as shown in Fig. 2c, d. Unless otherwise specified, negative charge patterns are chosen in the following experiments, owing to their long life time, than positive charge patterns at a given temperature.

**Fig. 3:**
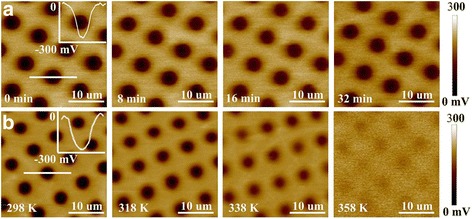
Surface potential images of patterned charges on ultrathin PS films with a thickness of 6 nm **a** with time extension under a constant temperature of 318 K and **b** under different temperatures for 3 min

The in situ surface potential images corresponding to electric charges with time extension or against temperature increase are recorded by EFM (Fig. 3). Accordingly, the charge dissipation is slow at low temperature, while it is accelerated upon the increase of temperature. At a relatively high temperature, e.g., 358 K, the charges are mostly dissipated in a few minutes, along with the disappearance of surface potentials.

**Fig. 4:**
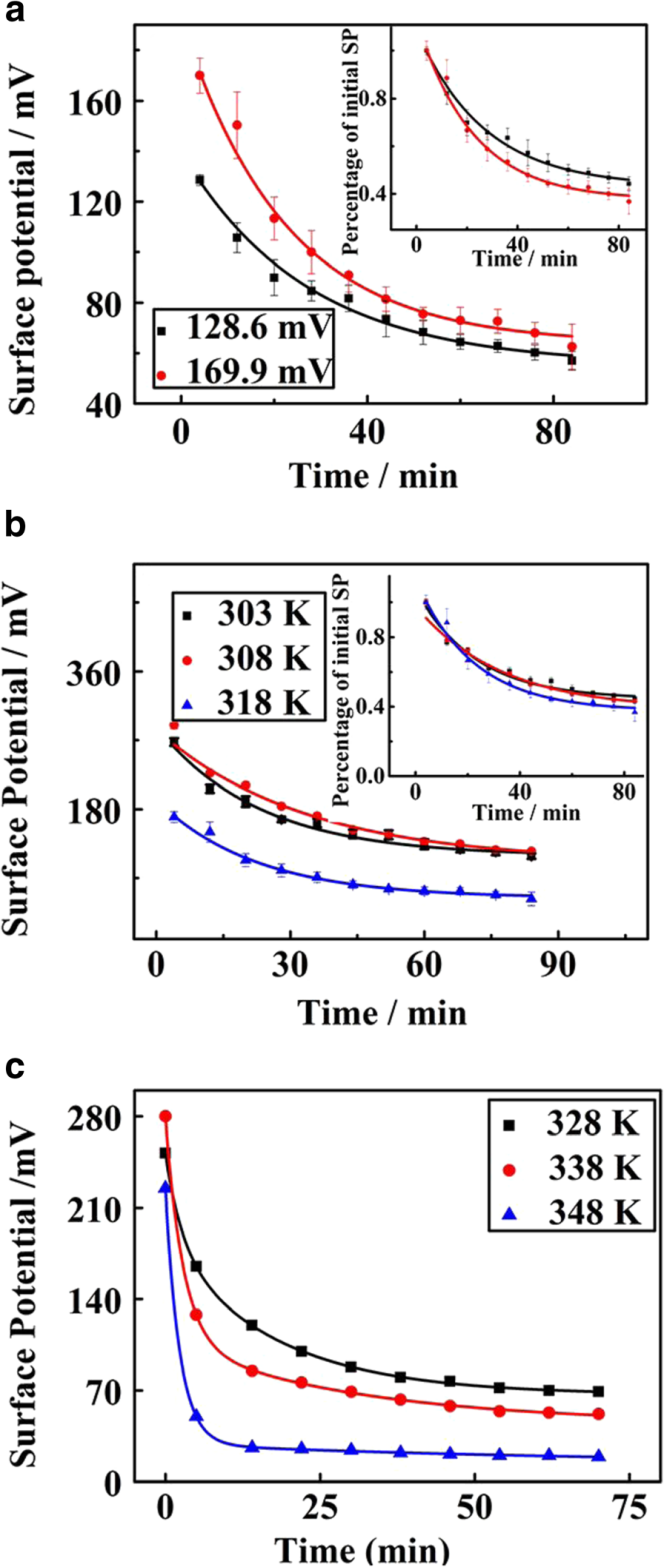
Time-elapsed isothermal discharging tendencies of ultrathin and normal thin PS films at different constant temperatures. **a** SPD of ultrathin PS film (6 nm) at 318 K with different initial surface potentials. **b** SPD of ultrathin PS film (6 nm) at 303, 308, and 318 K. **c** SPD of normal thin PS film (20 nm) at 328, 338, and 348 K. The normalized SPD with surface potential at 0 time as the reference is shown in the *inset*

The charge decay behavior referring to chain relaxation dynamics is quantitatively evaluated. As previously demonstrated, polymer chains in the ultrathin films begin to relax and loosen once the temperature is beyond a threshold, which accordingly accelerates the discharging behavior [36]. At a moderate temperature of 318 K which may not induce local dewetting [36], typical time-lapse isothermal SPD curves of an ultrathin PS film with the thickness of 6 nm are summarized in Fig. 4a. The discharging has a similar decreasing tendency with changing the initial surface potential. The charge decay is fast at the early stage yet tends to reach an equilibrium state over time. The overall characteristic decay time is estimated as tens of minutes. Notably, the discharging behavior begins at much lower temperature (318 K) than the bulk *T*_g_ or *T*_g_ of normal thin PS films, which indicates that local movement of polymer chains occurs much earlier. This phenomenon is also confirmed by other reported researches [18, 22, 41].

The temperature dependence of the discharging behavior is crucial to ultrathin film relaxation properties, especially at the segmental level. The discharging behavior is closely related to the polymer chain mobility, which are intensified under temperature stimulation especially for ultrathin films. Fig. 4b illustrates the isothermal SPD versus time at different constant temperatures of 303, 308, and 318 K for ultrathin PS film (6 nm). The inset of normalized results shows that the charge dissipation tendencies are nearly the same at 303, 308, and 318 K. However, as to normal thin PS films (more than 10 nm), higher temperature leads to sharper decay behaviors. Moderate temperatures of 328, 338, and 348 K are chosen for normal thin PS films of 20 nm, and the isothermal SPD curves over time at different temperature are illustrated in Fig. 4c. The charge decay behavior is accelerated under a higher temperature, which is caused by the intensification of polymer chain mobility under temperature stimulation.

**Fig. 5:**
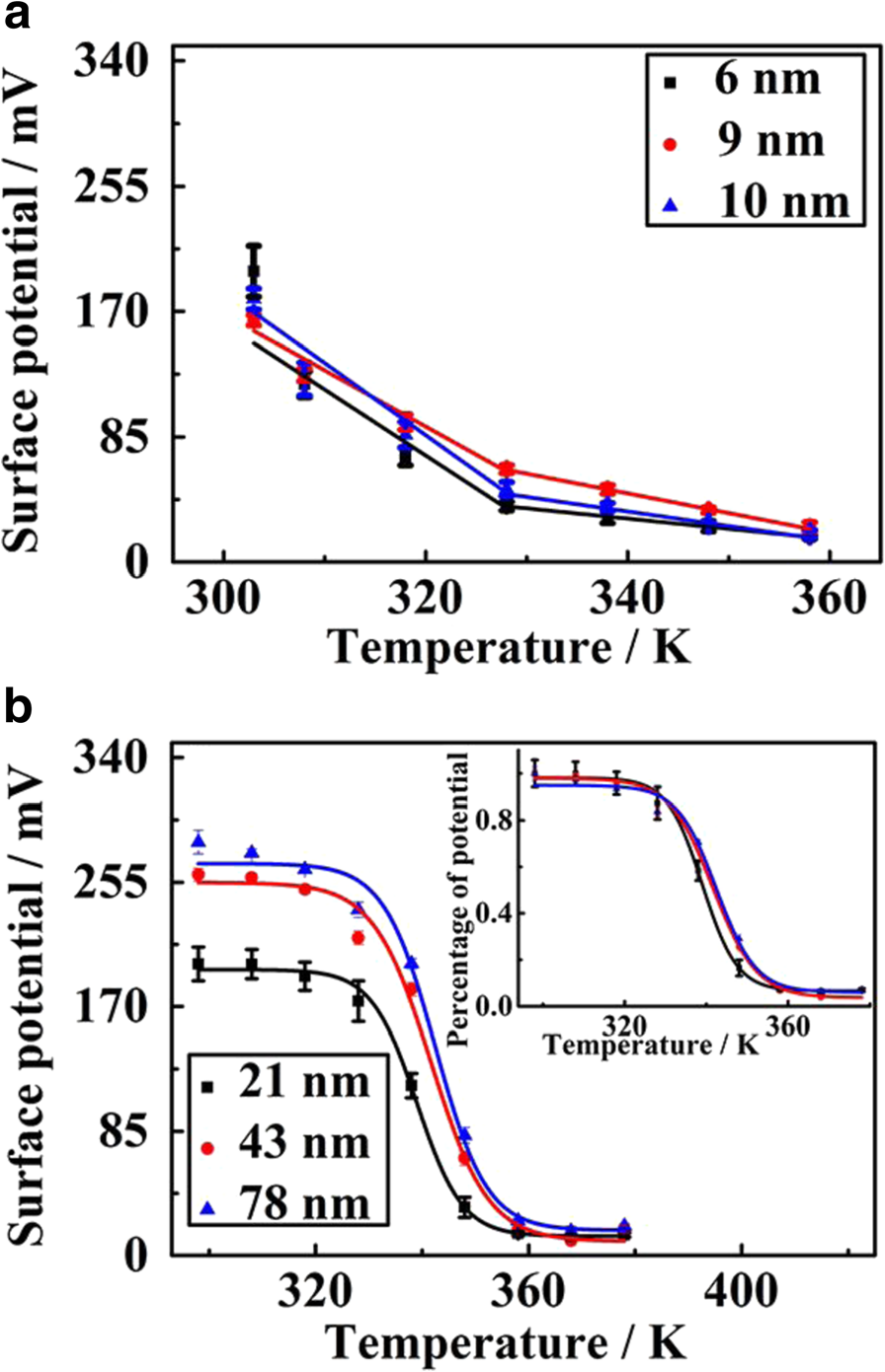
Temperature-dependent SPD curves of **a** ultrathin PS films with different thickness of 6, 9, and 10 nm and **b** normal thin PS films with thicknesses of 21, 43, and 78 nm

The temperature-dependent SPD curves for ultrathin PS films with different film thicknesses are illustrated in Fig. 5a. The discharging behaviors are quite similar when ultrathin PS film thickness reduces from 10 to 6 nm. Both linear and very sharp decay tendencies are obtained when the temperature increases from room temperature (298 K) to 328 K. With temperature continuously increasing, the sharp discharging behaviors tend to be gentle until patterned charges are nearly exhausted. According to the previous studies, the interface layer which is near the substrate could be negligible [11, 18], while the top layer and middle layer are regarded as the free surface layer and bulk layer [42], respectively. Polymer chains on the top surface layer have larger free space and higher mobility, which is much more active and independent of the bulk-like layer [10, 35, 39, 43, 44]. Hence, there is fast dynamics near the free surface compared to normal thin films and bulk matrix [13, 23, 45, 46], and most charges dissipate very quickly at the beginning stages. However, with the continuous dissipation of patterned charges, a reduction in the driving forces for the segmental relaxation could lead to a reduced discharging behavior, which conversely weakens the discharging behavior.

The discontinuous changes in the physical properties are always regarded as the glass transition temperature. For example, similar researches have proved that the temperature at the discontinuous changing of thermal expansivity or fluorescence intensity is regarded as the *T*_g_ [4, 18, 22, 47–50]. The transition temperatures in Fig. 5a are regarded as the *T*_g_ for ultrathin PS film with different film thicknesses. However, the *T*_g_ (328 K) in Fig. 5a is almost independent of thickness for ultrathin PS films, when the thickness is less than 10 nm. This result is also consistent with previously reported researches [18]. The two-layer model also indicates that the free surface layer (which is also regarded as liquid-like layer) exists when the thickness is below 10 nm, and it is independent of the overall film thickness [51]. Therefore, the film thickness of ultrathin PS films shows nearly independent of the discharging behaviors [22].

The observation of film thickness independences of *T*_g_ in ultrathin PS films is abnormal compared to reported results of normal thin polymer films, including PS thin films [4, 7, 32]. We then conduct experiments to identify the *T*_g_ of normal thin PS films, and the results are shown in Fig. 5b. Charge decay tendencies of normal thin PS films are well-defined exponential curves. A slow decay is observed at lower temperature, which is supposed to be related to confined movement of polymer segments. As the temperature increases, the movements of molecular chains are intensified, resulting in a sharp decay over the temperature range from 323 to 358 K. When the temperature further increases above 358 K, the SPD curves remain constant, which should be related to that the polymer main chains completely relax and trapped charges exhausted.

The SPD curves could be fitted as an exponential equation, in which the surface potential is as a function of temperature. The *T*_g_ of normal thin PS films could be calculated by the following equation:

1 $$ \varphi ={\varphi}_{\mathrm{r}}+\frac{\varphi_0-{\varphi}_{\mathrm{r}}}{1+{e}^{\left( T- Td\right)/ dT}} $$

Where *φ* is the measured surface potential of the thin PS film, *φ*_0_ is the initial surface potential (the patterned charges), and *φ*_r_ is the residual potential when the discharging curves approaching flat. *T* is the applied temperature on the PS films, and *dT* is the temperature width of glass transition region. *T*_*d*_ is the *T*_g_ that related to film thickness, at which charge decay is fastest.

The results in Table 1 show the calculated *T*_g_ shifts with decreasing film thickness, which are far lower than the *T*_g_ of the bulk matrix. The *T*_g_ of bulk PS film in this work is estimated to be 363 K using the differential scanning calorimetry (DSC). However, when the film thickness falls below 10 nm, the *T*_g_ remains constant and is also far lower than the one for normal thin PS films and bulk material.

**Table 1:** The *T*_g_ of PS films with various film thicknesses

Film thickness/nm	78	43	22	10	9	6
Transition temperature (*T*_g_/K)	343	342	339	328	328	328

For normal thin films, the structural relaxations, which are associated with a variety of small dynamics, is influenced by the interfacial and size confinement effects [22, 52]. However, it is regarded that there are only free surface layers and bulk-like layer for ultrathin polymer films [19], which results in weak interactions between the polymer chains.

**Fig. 6:**
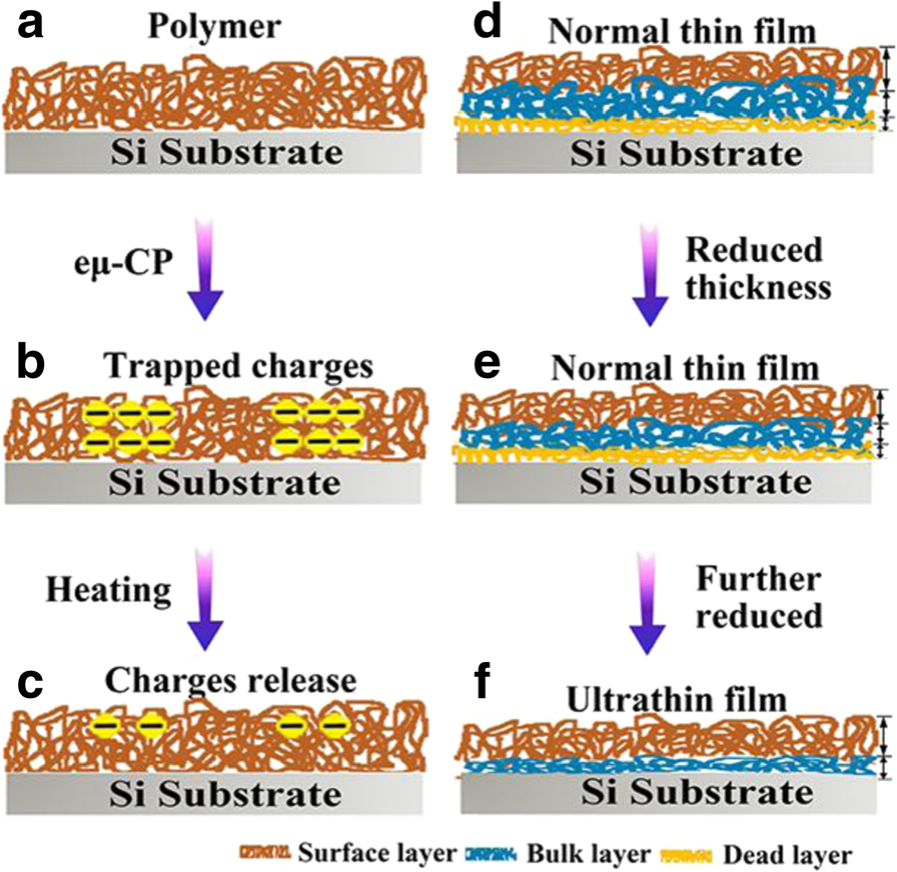
Diagram of patterned charges characterized polymer chains’ mobility and film thickness dependence of the *T*_g_ in thin and ultrathin polymer films. **a** Initial topographic polymer film, **b** selectively charged polymer film, **c** charges release, **d**, **e** film thickness dependent on local relaxation dynamics of normal thin films, **f** and ultrathin films

In order to clearly interpret the polymer chain mobility and the discharging behavior with film thickness decreasing, a schematic illustration is proposed as shown in Fig. 6. The discharging of patterned charges is closely related to the polymer chain’s mobility. The SPD tendencies are monitored in situ using EFM, as shown in Fig. 6a–c, and the relaxation dynamics and *T*_g_ could be estimated.

Two model theories are proposed to explain the phenomenon of the *T*_g_ depression with film thickness reducing. It is regarded that the dead layer in the three-layer model has almost no mobility [22]. Therefore, the shift of *T*_g_ is contributed both by surface layer (*T*sur*f* g) and bulk layer (*T*bulk g), in which the surface layer is thought as a region of enhanced mobility [28, 29]. Polymer chain ends at the air-polymer interface tends to move toward the surface, which leads to the increase of free volume and acceleration of chain mobility [30]. Therefore, the *T*surf g of surface is much lower than the *T*bulk *g* [38, 40]. The film thickness dependence of the *T*_g_ is illustrated as following [18]:

2 $$ {T}_g=\left\{{}_{{}^{T_g^{surf}\; for\; D< A}}^{{}^{\frac{1}{D}\left[ A{T}_g^{surf}+\left( D- A\right){T}_g^{bulk}\right]\; for\; D\ge A}}\right. $$

Where *D* is the total thickness of the films and *A* is the surface thickness (free surface layer) of polymer film.

Therefore, when the film thickness decreases, the relative fraction of *T*surf g to total *T*_g_ increases and leads to an overall decrease of *T*_g_ in normal thin PS film [30, 53], as shown in Fig. 6d, e. However, when film thickness continuously decreases below 10 nm, the dead layer disappears (Fig. 6f), the interaction between surface and bulk layer is weak, and the free surface layer contributes mostly to the depression of *T*_g_. As has been reported, the thickness of the free surface layer is assumed to be constant, which is independent of the thickness of films [18, 22]. Thus, the *T*_g_ of ultrathin PS film keeps constant.

The employment of the charge patterns as a more sensitive indicator to directly monitor the relaxation behavior could be feasible and precise, since the signals can be amplified and the transition points can be easily observed. However, it is worth noting that there is still some controversy about the relaxation dynamics and *T*_g_ of ultrathin polymer films. More challenging and rigorous studies should be conducted to quantitatively calculate the *T*_g_ of ultrathin polymer films.

## Conclusions

In summary, the relaxation properties and *T*_g_ of ultrathin PS film are characterized by directly monitoring the properties of the charge decay. When film thickness falls below 10 nm, linear discharging behaviors are obtained, and the *T*_g_ of ultrathin PS films is clearly independent of film thickness. This phenomenon is greatly different from that of the normal thin PS films, in which the *T*_g_ depresses with decreasing film thickness. To sum up, the discharging behavior of patterned charges provides a more precise approach to directly observe the relaxation dynamics and detect the *T*_g_ both for ultrathin and thin PS films. These results could be beneficial for understanding the relaxation dynamics of ultrathin polymer films, especially when the glass transition is considered.

## Abbreviations

AFM: Atomic force microscopy
DSC: Differential scanning calorimetry
EFM: Electric force microscopy
e-*μ*CP: Electric microcontact printing
LFM: Lateral force microscopy
PDMS: Polydimethylsiloxane
PS: Polystyrene
SPD: Surface potential decay
SVM: Scanning viscoelasticity microscopy
*T*_*g*_: Glass transition temperature
*T*surf g: Glass transition temperature at surface
